# Considerations on the Systematics of Neotropical Pierina, with the Description of Two New Species of *Phulia* Herrich-Schäffer from the Peruvian Andes (Lepidoptera: Pieridae, Pierinae, Pierini)

**DOI:** 10.1007/s13744-022-00999-y

**Published:** 2022-11-15

**Authors:** Tomasz W. Pyrcz, Keith R. Willmott, Gerardo Lamas, Pierre Boyer, Klaudia Florczyk, Christer Fåhraeus, Oscar Mahecha, José Cerdeña, Artur Mrozek, Jackie Farfán, Anna Zubek

**Affiliations:** 1 Dept of Invertebrate Evolution, Institute of Zoology and Biomedical Research, Jagiellonian Univ, Kraków, Poland; 2 McGuire Center for Lepidoptera and Biodiversity, Florida Museum of Natural History, Univ of Florida, FL Gainesville, USA; 3 Museo de Historia Natural, Univ Nacional Mayor de San Marcos, Lima, Perú; 4 Le Puy Sainte Réparade, France; 5 Nature Education Centre of the Jagiellonian Univ, Kraków, Poland; 6 Dept of Biology, Lund Univ, Lund, Sweden; 7 Grupo en Biogeografía y Ecología Evolutiva Neotropical BEEN, Univ Distrital F.J.C./Univ Incca de Colombia, Bogotá, Colombia; 8 Univ Nacional de San Agustín de Arequipa, Museo de Historia Natural, Escuela de Biología UNSA, Arequipa, Perú

**Keywords:** *P. stoddardi* n. sp., *P. phantasma* n. sp., COI barcoding, Cordillera Negra, Male and female genitalia, Puna, Venation

## Abstract

A comparative analysis of high-Andean Pierina was carried out, including a total of 25 species. Based on morphological evidence, with an emphasis on venation and genitalia and molecular data, using three genetic markers, we confirm the recent subjective synonymy of the generic names *Tatochila* Butler, 1870, *Piercolias*, Staudinger, 1894, *Hypsochila* Ureta, 1955, *Infraphulia* Field, 1958, *Pierphulia* Field, 1958, and *Theochila* Field, 1958 with *Phulia* Herrich-Schäffer, 1867. Two new species are described, namely *Phulia stoddardi* Pyrcz & Cerdeña n. sp., from the Andes of Central Peru, which occurs at an unusually high altitude of close to 5000 m a.s.l. in dry puna habitat, and *Phulia phantasma* Lamas, Willmott & Boyer n. sp., from dry montane forests in northern Peru and southern Ecuador. An overview of high-elevation butterflies is presented, with some discussion on adaptations to this environment.

## Introduction

As a general rule, species diversity decreases with elevation, with many hypotheses proposed to explain this pattern (Hortal et al. 2013), which is seen in insects, including Lepidoptera, and butterflies in particular (Lawton et al. 1987; McCoy 1990). For individual lineages, however, there are of course exceptions, and the diversity of certain groups is highest at middle elevations, up to the timberline around 3000 m a.s.l. (Brehm et al. 2003; Pyrcz 2004; Pyrcz et al. 2009). However, invariably, once the forest-grassland ecotone is crossed, in both temperate and tropical areas, species diversity dramatically decreases, and it continues to fall with increasing elevation (Pyrcz 2010). Close to the snowline, situated generally around 5000 m a.s.l., very few butterflies exist.

Butterflies occur at or above 5000 m a.s.l. above the sea level in just two regions of the world. In central Asia, several species of Papilionidae, Pieridae, Lycaenidae, Nymphalidae, and Hesperiidae have been reported from altitudes above 5000 m a.s.l. (Tshikolovets 2005; Acharya and Vijayan 2015). Some may be classed as migrants, occasionally observed at elevations well above their normal reproductive areas, such as the renowned migrant *Vanessa cardui* (Linnaeus, 1758). There are, nevertheless, some Asian species whose entire life cycle occurs within extremely high elevations, mostly in the genus *Parnassius* Latreille, 1804 (Papilionidae), and it is among them that altitude record-holders can be found, including *P. charltonius* Gray, [1853] (5400 m a.s.l.), *P. stoliczkanus* C. Felder & R. Felder, 1865 (5500 m a.s.l.), *P. acdestis* Grum-Grshimaïlo, 1891 (5600 m a.s.l.), *P. maharaja* Avinoff, 1916 (5700 m a.s.l.), *P. acco* Gray, [1853] (5700 m a.s.l.) and *P. simo* Gray, [1853] (5700 m a.s.l.). A couple of members of the lycaenid genus *Polyommatus* Latreille, 1804 are known to occur above 5000 m a.s.l., including *P. stoliczkana* (C. Felder & R. Felder, 1865) and *P*. (*Agriades*) *lehanus* Moore, 1878 (5600 m a.s.l.). Finally, in central Asia, two groups of Pieridae can be found with some frequency above 5000 m a.s.l., including Coliadinae, *Colias stoliczkana* Moore, 1878 (5600 m a.s.l.)*, C. fieldii* Ménétriés, 1855 (5400 m a.s.l.), *C. ladakensis* C. Felder & R. Felder, 1865 (5300 m a.s.l.) and *C. thrasibulus* Fruhstorfer, 1910 (5600 m a.s.l.), as well as Pierinae, (*Pontia*) *Baltia butleri* (Moore, 1882) (5500 m a.s.l.), and *Pontia* (*Pontia*) *callidice* (Hübner, [1800]) (5600 m a.s.l.). Some Hesperiidae of the genus *Pyrgus* Hübner, [1819], and Satyrinae of the genera *Paralasa* Moore, 1893, *Karanasa* Moore, 1893 and *Paroeneis* Moore, 1893 can be found at elevations approaching 5000 m a.s.l. (Tshikolovets 2005). Nonetheless, most, if not all, of the above species have rather broad elevational ranges and also occur at elevations down to 4000 m a.s.l.

The situation is mirrored in the high Andes, notably in the Altiplano of Peru, Bolivia, and Chile. Contrary to Asia, there are no high-elevation Papilionidae, but several species of Hesperiidae (*Pyrgus*, *Hylephila* Billberg, 1820), Heliconiinae (*Yramea* Reuss, 1920), Satyrinae (*Argyrophorus* Blanchard, 1852, *Faunula* C. Felder & R. Felder, 1867), and Lycaenidae (*Itylos* Draudt, 1921, *Paralycaeides* Nabokov, 1945, *Pseudolucia* Nabokov, 1945) that are found at 4500–4700 m a.s.l. (Cerdeña et al. 2014; Despland 2014). The picture as regards to Pieridae is somewhat different. Of the Coliadinae, two species (*Colias flaveola weberbaueri* Strand, 1912, and *C. euxanthe hermina* (Butler, 1871)) can be observed near 5000 m a.s.l., even if their habitats are generally situated a couple of hundred meters below, but at least 15 species within Pierinae have been recorded at elevations approaching 5000 m a.s.l. (Despland et al. 2012). These Pierinae species have until recently been classified within six genera, most of which, as discussed below, are now regarded as junior subjective synonyms, namely *Phulia* Herrich-Schäffer, 1867, *Pierphulia* Field, 1958, *Infraphulia* Field, 1958, *Piercolias* Staudinger, 1894, *Hypsochila* Ureta, 1955, and *Tatochila* Butler, 1870, although the latter two genera have also been regarded as containing some species down to sea level in Patagonia. Among these, only the former genus *Piercolias* is confined to elevations around 5000 m a.s.l., representing one of the highest flying butterflies in the world (Oram 2005). The holotype of *Piercolias forsteri* Field & Herrera, 1977 was collected at 5100 m a.s.l. on the slopes of Cerro Illimani in Bolivia. The record-holder in altitude among Neotropical butterflies is, however, *Pierphulia isabelae* Field & Herrera, 1977, whose holotype was collected by José Herrera on the volcano Ojos del Salado in Chile at 5400 m a.s.l. (Field and Herrera 1977).

The taxonomy of high-altitude Andean Pieridae was revised by Field (1958), who proposed two new genera and diagnosed all the genera based on morphology (venation, legs, and male and female genitalia). His arrangement was, until recently, considered valid and unchallenged. Shapiro et al. (2007) studied the relationships between high-elevation Andean pierines and *Baltia* based on two molecular markers (COI and COII), obtaining a monophyletic clade including *Pierphulia*, *Infraphulia*, *Pierphulia*, *Tatochila*, and *Hypsochila*, and pointed out that *Baltia* is not a close relative of these. Wahlberg et al. (2014) proposed a hypothesis of the phylogeny of Pieridae based on five molecular markers, in which they confirmed the monophyly of the above genera. They also included the Brazilian monobasic *Theochila* Field, 1958, which was placed in a clade with *Tatochila* and *Hypsochila*, sister to a clade including four exclusively high-elevation genera, even if all these genera appeared to be very closely related.

In a recent paper, Zhang et al. (2021) used all protein coding genes retrieved from whole-genome shotgun sequences to infer the phylogeny of various butterfly lineages, proposing many new combinations and generic synonyms, and describing several new genera and higher taxa. In their conclusions, with which we concur with some exceptions discussed below, the genera *Tatochila*, *Piercolias*, *Hypsochila*, *Theochila*, *Pierphulia*, and *Infraphulia* were all treated as junior subjective synonyms of *Phulia* Herrich-Schäffer. Also, in the same paper, for the first time, molecular data were included for *Piercolias* and the monotypic *Reliquia* Ackery, 1975, endemic to the Colombian Sierra Nevada de Santa Marta, with the latter newly regarded as a junior subjective synonym of *Pontia* [Fabricius], 1807.

The discovery of two new species described in this paper, and the uncertainty regarding their generic classification, inspired us to revisit the systematics of high-altitude Neotropical Pierina (with the exception of the loosely related genus *Leptophobia*, Butler, 1870, which comprises several mid to high-altitude Andean species, belonging to the sister clade of Pierina including non-neotropical genera, such as *Pieris* Schrank, 1801), with the benefit of both morphological and molecular data and a more comprehensive taxon sample than included in previous studies.

## Material and methods

### Material

Sampling was carried out with a standard entomological hand-net during several field expeditions co-organized by the Museo de Historia Natural de la Universidad Nacional de San Agustín (MUSA) in Arequipa, Peru in June 2019, and the Nature Education Centre of the Jagiellonian University (CEP-UJ)—formerly Zoological Museum of the Jagiellonian University (MZUJ). Fieldwork was also conducted throughout Peru by GL and collaborators (Museo de Historia Natural de la Universidad Nacional Mayor de San Marcos, Lima, Peru (MUSM) over the last 50 years. Material was set and examined, and genital dissections were made mainly in (MUSA) and CEP-UJ, including the collection of Pierre Boyer (PBF). Additional material was examined in the McGuire Center for Lepidoptera and Biodiversity, Florida Museum of Natural History, University of Florida, Gainesville, USA (FLMNH), and the collection of Maurizio Bollino, Lecce, Italy (MABO).

### Morphology

Male and female abdomens were soaked in 10% KOH solution for 5–10 m a.s.l. min and then cleaned out of soft tissue in water in order to expose genital parts. Female abdomens were stained in chlorazole black to better visualize soft genital tissues. Dissected genitalia were dehydrated by using ethanol 90% and 95% solutions. Head microstructures were examined under an Olympus SZX9 stereo-microscope. Adults were photographed with an Olympus E-500 digital camera and plates were composed with Adobe PhotoShop 8. Nikon digital camera DS-Fi1 and Olympus SZX9 stereo-microscope were used for taking pictures of the dissections, which were then processed in Combine ZP and Corel PHOTO-PAINT X3 programs to enhance clarity and improve quality. Genital dissections were kept in glycerol vials pinned under corresponding specimens. Genital terminology largely follows Razowski (1996) and Klots (1956). The following abbreviations were used: FW, forewing; HW, hindwing; D, dorsum; V, venter.

### Molecular analysis

#### Extraction and amplification of DNA

For molecular analysis, two legs were removed from each of the 52 selected specimens, representing all six previously recognized genera of Pierina that occur in high-Andean habitats (*Phulia*, *Infraphulia*, *Piercolias*, *Pierphulia*, *Tatochila*, and *Hypsochila*), along with the southeast South American *Theochila.* Legs were detached and preserved in 1 ml of ethanol, prior to mounting of the voucher specimen. DNA was extracted using Macherey–Nagel’s Nucleospin Tissue extraction kit, following the manufacturer’s protocol. Amplification of three separate gene regions, one mitochondrial, and two nuclear was performed in a 20 ul volume. The following primers were used: LCO1490 and HCO2198 (Folmer et al. 1994) for COI (Cytochrome c oxidase subunit I), RpS5f and RpS5r for RpS5 (Ribosomal Protein S5), and Frigga and Burre (Wahlberg and Wheat 2008) for GAPDH (glyceraldehyde-3-phosphate dehydrogenase). The following PCR cycling profile was applied for COI: 95 °C for 5 min, 40 cycles of 94 °C for 30 s, 50 °C for 30 s, 72 °C for 1 min 30 s, with a final extension period of 72 °C for 10 m a. s. l.in; and for both nuclear genes: 95 °C for 5 min, 40 cycles of 94 °C for 30 s, 55 °C for 30 s, 72 °C for 1 min 30 s, and a final extension period of 72 °C for 10 m a.s.l. in PCR products were sent for purification and sequencing to Macrogen Europe (Amsterdam, Netherlands). Additional sequences of 13 related species were imported from GenBank and BOLD System databases: *Phulia nymphula* (Blanchard, 1852)*, Pierphulia rosea* (Ureta, 1956)*, Infraphulia ilyodes* (Ureta, 1955), *Tatochila autodice* (Hübner, 1818), *Tatochila mercedis* (Eschscholtz, 1821), *Tatochila homoeodice* (Paravicini, 1910), *Tatochila theodice* (Boisduval, 1832), *Theochila maenacte* (Boisduval, 1836), *Hypsochila wagenknechti* (Ureta, 1938), *Hypsochila microdice* (Blanchard, 1852), with *Ganyra josephina* (Godart, 1819), and *Ascia monuste* (Linnaeus, 1764) as outgroups*.* Sequences were edited and aligned manually using Bioedit version 7.0.9.0. (Hall 1999). Two datasets were prepared, the first one with COI sequences (74 samples, 612 bp) and the second one including COI, GAPDH, RpS-5, with 60 samples, and 1892 bp in the combined dataset.

#### Phylogenetic inference

Analyses were carried out for the data set of the three genes combined, as well as for the separate COI data set. Phylogenetic trees were inferred using maximum likelihood (ML). Model selection was determined according to the hierarchical likelihood ratio test as implemented in ModelTest 3.06 (Posada and Crandall 1998), with the starting tree obtained by maximum parsimony to estimate model parameters. The data were analyzed in MEGA X (Kumar et al. 2018) with the GTR + G + I model of sequence evolution and 10,000 bootstrap replicates to estimate branch support.

We performed a divergence time estimation using the StarBEAST 2 v.0.15.5 package (Ogilvie et al. 2017) available in BEAST2 suite v. 2.6.3 (Bouckaert et al. 2019), which implements the Bayesian multispecies coalescent method to infer calibrated species trees (Ogilvie et al. 2017). Substitution models were inferred for each locus with Partitionfinder v.0.1 (Lanfear et al., 2012; Bouckaert & Drummond, 2019). An uncorrelated relaxed clock was used for all loci. We assigned to the mitochondrial COI locus a gene ploidy of 0.5 and to the GAPDH-RpS5 nuclear loci a gene ploidy of 2.0. The Birth–Death model (Gernhard 2008) was used with GrowthRate prior as log-normal (*M* = 5, *S* = 2) and relative DeathRate as uniform in [0, 1], while the popMean prior was set to log-normal (*M* =  − 7, *S* = 2) (Jones et al. 2015). The MCMC chains were run for 70 m a.s.l. million generations and sampled once every 1000 generations. Three independent runs were started and combined with LogCombiner v. 2.5.2 (Bouckaert et al. 2019). The posterior distribution of trees from the analysis was then summarized in TreeAnnotator v. 2.5.2 (Bouckaert et al. 2019) with a selected burn-in of 25% to build the maximum clade credibility tree, and posterior probabilities (pp) were calculated as branch support. Chain convergence was further assessed with Tracer v.1.6 (Rambaut et al. 2014) to confirm sufficient effective sampling size (ESS > 200) (BEAST Developers 2017). Node support is defined as low/weak (pp < 0.50), moderate (pp: 0.50–0.84), high (pp: 0.85–0.94), or strong (pp ≥ 0.95). The trees were visualized using Figtree v.1.4 (Rambaut 2009). To plot the ultrametric tree, the R package Strap (Bell & Lloyd 2015) was used, and the resulting trees were refined in Inkscape 0.92.3 (https://www.inkscape.org).

We used secondary calibrations to time-calibrate the species tree (see Peña et al. 2011; Chazot et al. 2019a, 2019b; Matos-Maraví et al. 2021). The selected calibration points were the crown age of the Pieridae at 76.9–15.9 Mya, and the maximum age of the clade Coliadinae + Pierinae at 66.7 Mya (Chazot et al. 2019a). Furthermore, we also constrained the root with the age of the hypothesized ancestral host plant of the Pierinae, with a maximum age corresponding to the crown age of Brassicaceae to 97 Mya (Foster et al. 2017; Chazot et al. 2019a).

## Results

### Taxonomy

#### *Phulia stoddardi* Pyrcz & Cerdeña n. sp.

Types: HOLOTYPE (male): PERU, *Ancash*: ruta Conococha – Ocros, Abra, 10°15′22″S,77°15′11″W, 4800 m, 11.vi.2019, J. Farfán and J. Cerdeña, [MUSM]; Paratypes (10 males and 4 females): 1 male and 1 female: same data as the holotype [MUSA]; 5 males and 2 females: Peru, Ancash, paso carretera Conococha–Ocros, 4800–4850 m a.s.l., 13.vi.2019, T. Pyrcz [CEP-UJ] (1 female prep. genit. 1751_18.07.2019/K. Florczyk; prep. mol. CEPUJ 20,190,713, prep. mol. CEPUJ 20,190,714); 4 males and 1 female: Ocros vers Huaraz km 27, 10°15′23″S,77°15′14″W, 4800 m, 11.vi.2019, P. Boyer leg., [PBF].

**Fig. 1:**
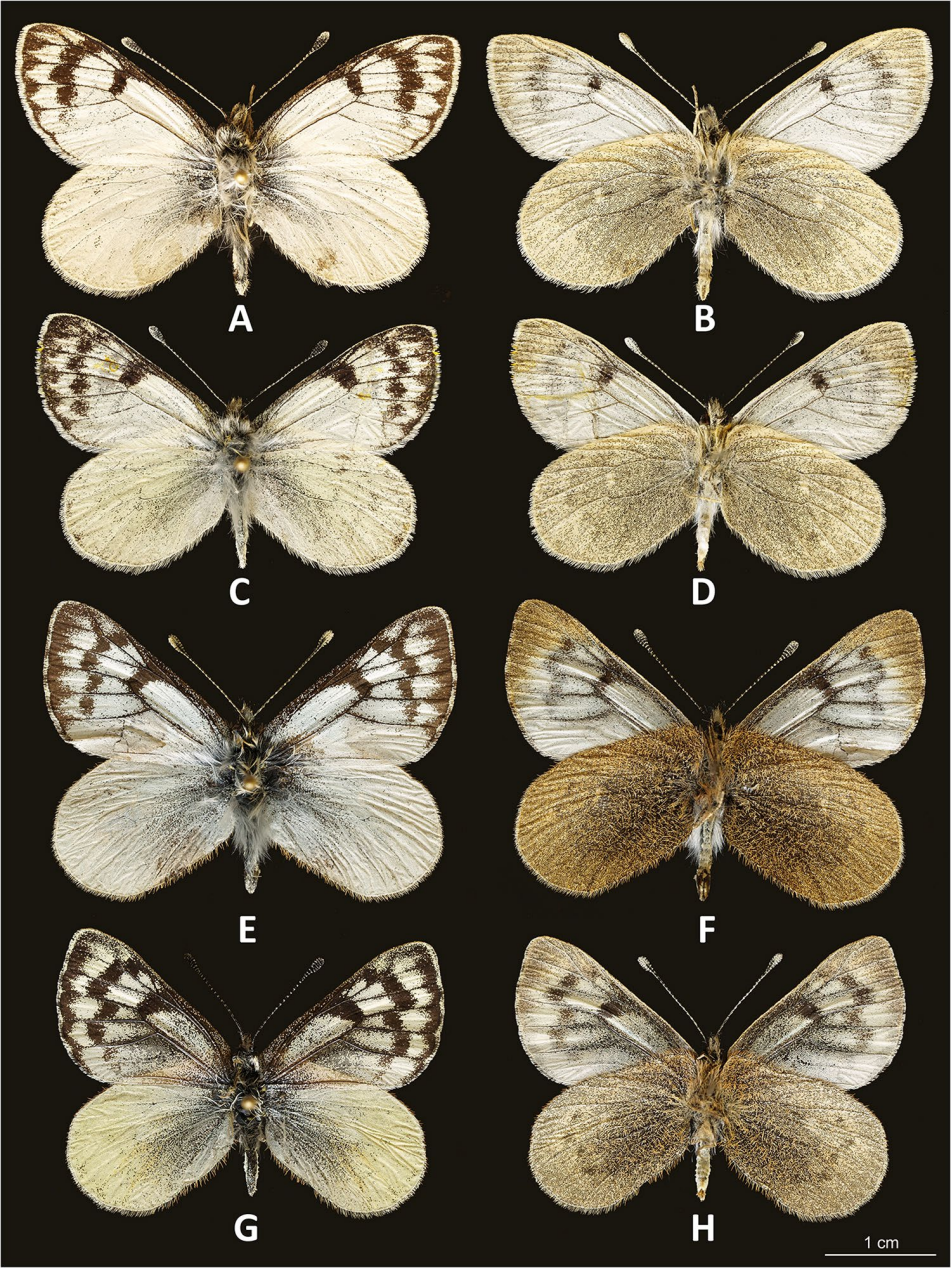
Adults. **A** *Phulia stoddardi* male holotype upperside; **B** *idem*, underside; C. *Phulia stoddardi* female paratype, upperside; **D** *idem*, underside; **E** *Phulia* (*Piercolias*) cf. *forsteri* male, upperside (Ticlio); **F** *idem*, underside; G. *Phulia* (*Piercolias*) cf. *forsteri* female upperside (Ticlio); **H** *idem*, underside

**Fig. 2:**
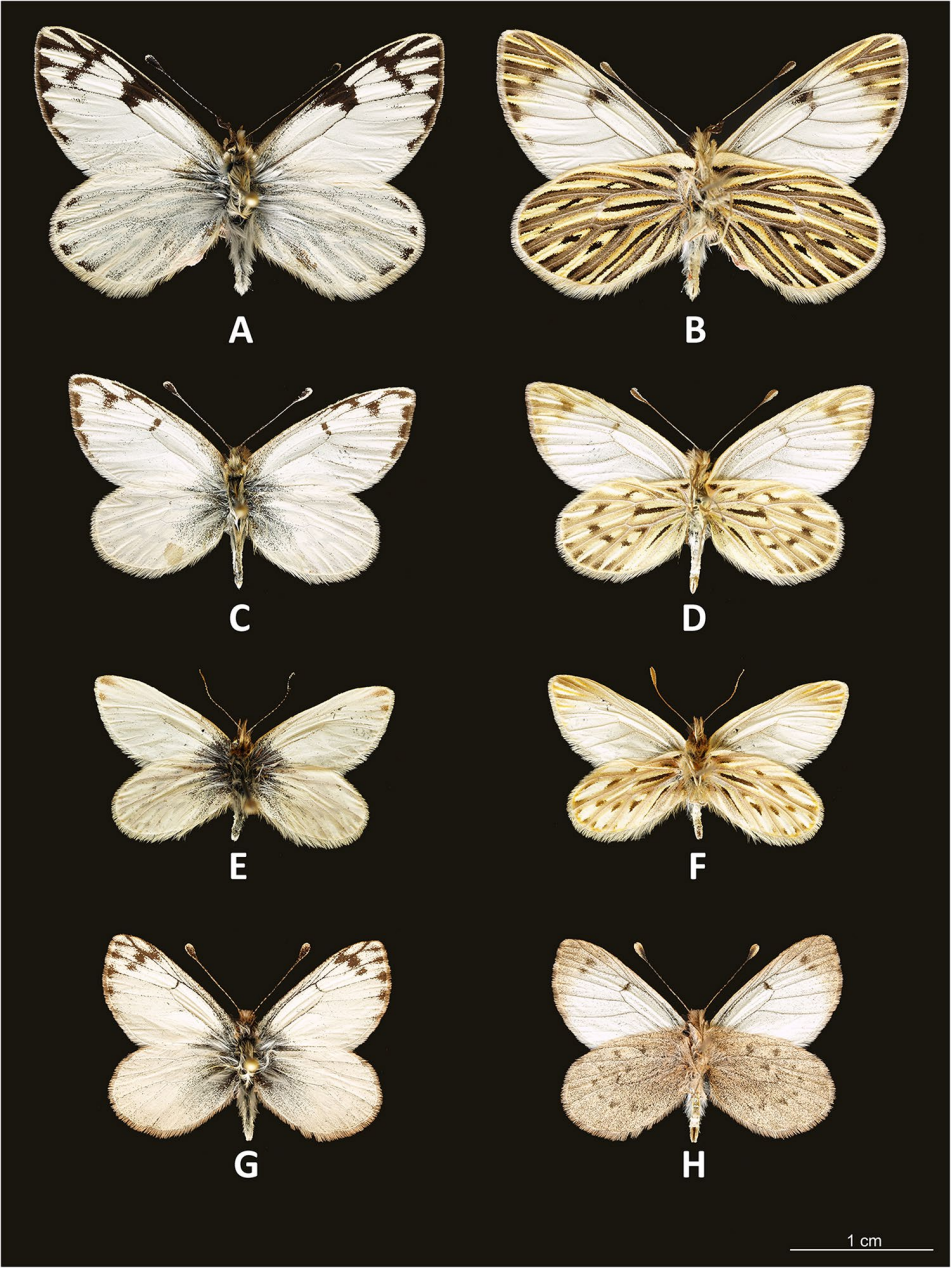
**A** *Phulia nymphula nymphula* male, upperside; **B** *idem,* underside; **C** *Phulia nannophyes nannophyes* male, upperside; **D** *idem*, underside; **E** *Phulia* (*Infraphulia*) *madeleinea* male, upperside; F. *idem*, underside; G. *Phulia* (*Pierphulia*) *nysias* male, upperside; H. *idem,* underside

*Diagnosis*: The new species is closely similar in wing size and shape to species previously assigned to the genus *Piercolias*, in particular *P.* cf. *forsteri* (Fig. 1E–H) from Ticlio, Lima, Peru, and *P. huanaco* (Staudinger, 1894), although the FW of *Phulia stoddardi* n. sp. is more elongated with a more convex outer margin. Also, the color pattern is similar to that of *P.* cf. *forsteri*, from which it differs in being lighter, with less black on the FWD, and in particular, by the row of subapical black spots which is parallel to the outer margin, whereas in other species previously assigned to *Piercolias* that same row is sharply displaced basally in cell M_3_-Cu_1_. The HWV color is similar to *Piercolias*, almost patternless but even darker, with a strong greenish suffusion as in *P.* cf. *forsteri*, in contrast to the dashed pattern of other high-elevation Pierinae, such as *Phulia* sensu stricto (Fig. 2A–D) and *Infraphulia* (Fig. 2E–F). The wing pattern of the species placed until now in *Pierphulia* (Fig. 2G–H) is, in this respect, intermediate, as it shows darker postdiscal dots on a uniform ground color.

**Fig. 3:**
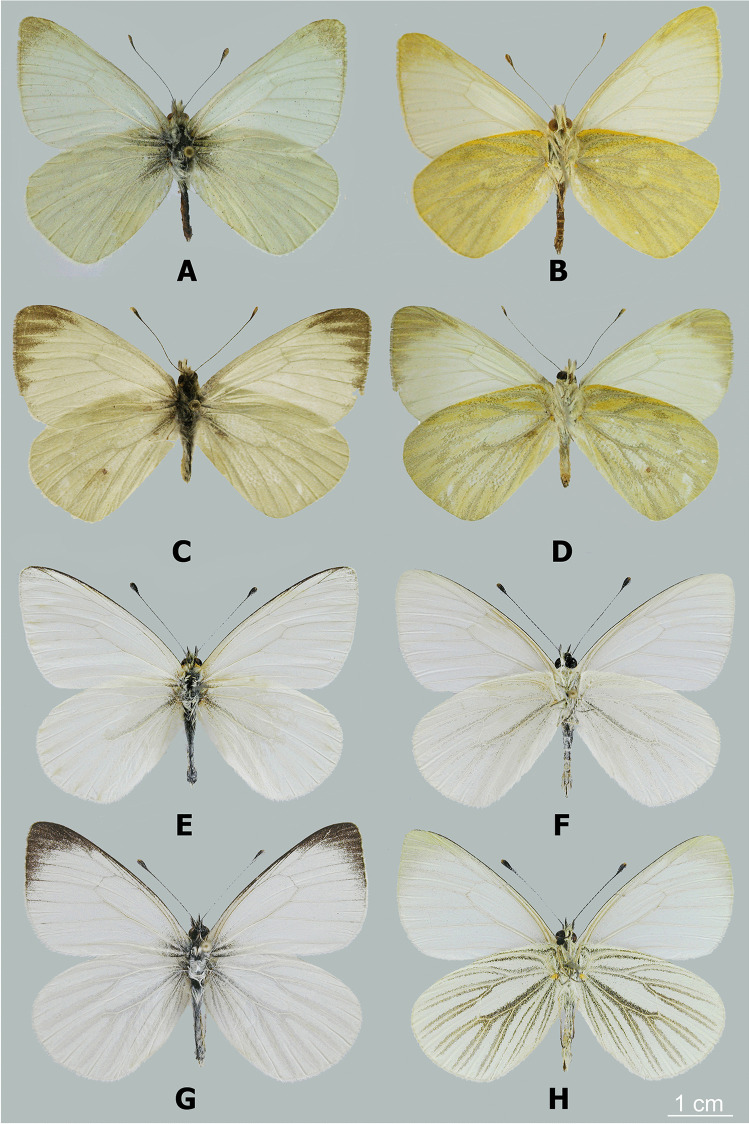
Adults: **A** *Phulia phantasma* male holotype, dorsal (Cerro Amancaes); **B** *idem*, ventral; **C** *P. phantasma* female paratype, dorsal (Llacanora); **D** *idem*, ventral; **E** *Phulia* (*Theochila*) *maenacte maenacte* male (Sao Francisco de Paula), dorsal; **F** *idem*, ventral; **G** *Phulia* (*Theochila*) *maenacte itatiayae* male (Itatiaya), dorsal; **H** idem, ventral

**Fig. 4:**
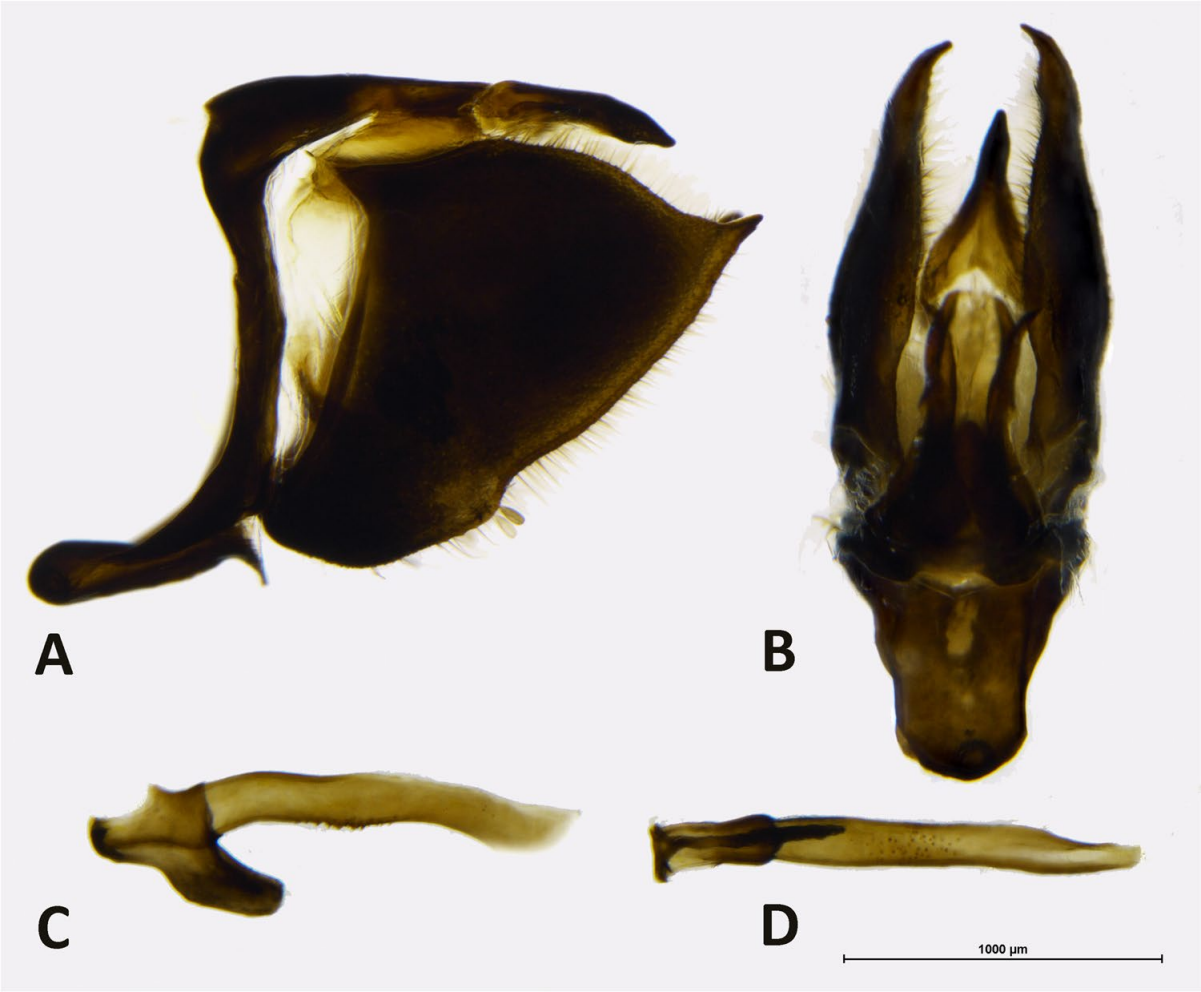
Male genitalia of *Phulia stoddardi* n. sp.: **A** lateral view; **B** vertical view; **C** aedeagus lateral view; **D** aedeagus vertical view

**Fig. 5:**
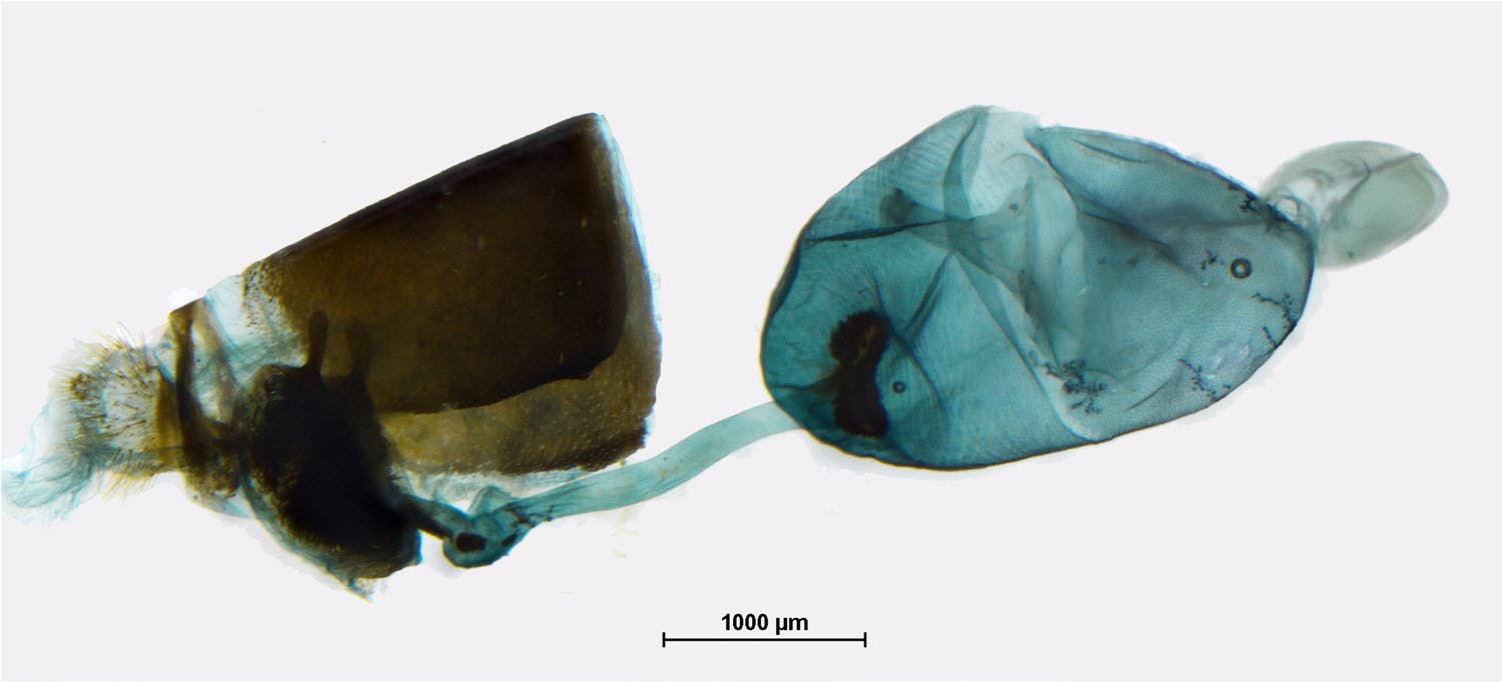
Female genitalia of *Phulia stoddardi* n. sp.: lateral view

*Description*: Adults: MALE (Fig. 1A–B): Head: eyes patchy chestnut and dark brown, naked; palpi 2.5 times the length of head, black, covered with milky white scales; antennae reaching 2/3 of costa, black covered intermittently with white scales, denser on ventral surface of spoon-shaped club. Forewing (length: 17–18 mm) elongated with a convex outer margin and a subacute apex, densely hairy in basal part; fringes long and white. Venation (Fig. 7) characterized by the common stem of forewing M_2_ and *R*_3+4_; *R*_2_ arising basally from the stem of R_3_, not from the same stem as in *Piercolias*, but similarly to *Phulia* and *Pierphulia*; hindwing M_1_ and M_2_ arising from the same stem, as in *Phulia* but contrary to *Piercolias*, *Pierphulia*, and *Infraphulia*. Hindwing oval, densely hairy in basal part; fringes long and white. FWD mostly snow white except for some black scales along costa, a black discal cell end patch, a row of subapical patches, parallel to outer margin, from costa to vein Cu_1_, and a row of marginal, roughly triangular patches from apex to vein Cu_2_, gradually smaller. HWD snow white, dusted with some black scales in basal and post-basal area. FWV predominantly snow white, dusted with cream yellow scales along costa, outer margin, apical and subapical area; a faint discal cell black patch; and a series of equally faint subapical patches in cells M_1_-M_2_ to M_3_-Cu_1_. HWV cream yellow liberally dusted with black scales, denser in basal and post-basal areas, with a series of faint postdiscal black dots, one in every cell. Male genitalia (Fig. 4): similar to other congeners. Uncus short, similar to species formerly placed in *Phulia*, considerably shorter than tegumen, slightly incised basally; tegumen smooth and thin, uncus short, similar to former *Phulia*, valva massive with a sharp distal tip, as in former *Piercolias*, aedeagus short and tubular with a serrate ventral surface and a prominent basal bulbous projection. FEMALE (Fig. 1C–D): sexual dimorphism is slight, the female is somewhat smaller (FW length 15–17 mm), with more prominent black pattern and on the upperside the ground color is richer, milky white. Female genitalia (Fig. 5): Papillae weakly sclerotized and setose; prominent posterior apophyses, genital plates, consisting of large, lateral pockets, inwardly densely setulose, long and scarcely sclerotized ductus bursae except for the initial part, with a prominent accessory pouch, a large corpus bursae with large, double star-like signa at the entrance of ductus and a rudimentary secondary bursa, similar to the species placed previously in *Piercolias*.

*Etymology*. This species is dedicated to Terry Stoddard, an amateur lepidopterist from the U.S.A., and co-author of many significant contributions to the knowledge of Nearctic butterflies, in recognition of his excellent collaboration over the years and for sharing his experience on high-elevation butterflies.

#### Bionomics and distribution

This species has been found so far only at the type locality at the highest part of the road from Conococha to Ocros, at 4800–4950 m a.s.l., on the northern side of the slopes (Fig. 13). Individuals were observed on a dry hillside almost devoid of any vegetation (Fig. 11A–B), except for some sparse “cushion” plants. Two species of *Senecio* (Asteraceae) were seen, one higher up on the mountain ridge, the other, lower, closer to a bog. Among cushion plants, three species of Asteraceae, *Werneria* sp., *Baccharis* sp., and *Chaetanthera* spp., were identified, as well as *Gentiana sedifolia* (Gentianaceae) (Fig. 11D–H). No Brassicaceae, a likely hostplant, were observed although, admittedly, due to time constraints, our search was not exhaustive. Individuals of *Phulia stoddardi* n. sp. remained inactive for most of the morning, until around 11 am when gusty winds stopped blowing. They were observed performing short patrolling flights, very low to the ground, and engaging in energetic interactions with passing individuals. After such interactions, they dropped to the ground and sought shelter or basked on stones with their wings wide open, touching the substrate, in a similar way as with other high-elevation Pierinae. Lateral basking, a posture common among *Colias* Fabricius, 1807, was not observed. When the temperature decreased, *P. stoddardi* n. sp. adults were observed seeking shelter by crawling under the rocks, presumably to avoid both freezing and desiccation. Adults were occasionally seen feeding on flowers, although at rest they were more frequently observed sitting on dry, sandy, and rocky slopes. Other species of butterflies seen in the area were exclusively pierids, including occasional passing *Colias euxanthe* C. Felder & R. Felder, 1865 and some *Phulia garleppi* Field & Herrera, 1977 (Fig. 11C), with the latter much more common around a bog situated approximately 150 m a.s.l. below the habitat of *P. stoddardi* n. sp.

### *Phulia phantasma* Lamas, Willmott & Boyer, n. sp.

[*Hypsochila* n. sp. Lamas (Lamas, 2004: 115, no. 315)]

*Types*: HOLOTYPE (male): PERU, *Ancash*, Cerro Amancaes, cerca Santo Toribio, [08°50′S,77°54′W], 3000 m a.s.l., 22.v.[19]80, G. Lamas, MUSM-ENT-006767, [MUSM]; PARATYPES (37 ♂ and 1 ♀): *Ancash*: 2 males: same data as holotype, MUSM-ENT-006759, 6763, [MUSM]; 2 males: [Cerro] Amancaes, N de Huaylas, [8°50′S,77°54′W], 2900 m a.s.l., 6.v.[19]79, V. Pacheco, MUSM-ENT-006762, 6768, [MUSM]; 1 male: same data, but 3000 m, 8.v.[19]79, MUSM-ENT-006758, [MUSM]; 1 male: Oriente de la Cordillera Negra, C[omunidad] C[ampesina] Shecta, 9°28′06″S,77°35′43″W, 4131 m, 05.ii.2012, B. Medina, [MUSM]; 6 males: road from Bamba, T. Pyrcz [CEP-UJ]; 1 male: same data [PBF]; 4 males: same data, J. Cerdeña & J. Farfan leg., [MUSA]; *Cajamarca*: 1 male: Cascas-Contumazá p[oste] k[ilometrique] 100, [7°24′35″S,78°48′4″W], 2750 m, 23.iii.2022, P. Boyer, [PBF]; 1 male: Cascas-Contumazá “borne kilometrique” 100, [7°24′66″S,78°48′076″W], 2650–2750 m, 19.vi.2018, [PBF]; 1 male: same data, [MUSA]; 6 males: Chilasque, 06°01′S,79°12′W, 1200 m, 13.vi.2000, G. Lamas, MUSM-ENT-006760, 6761, 6764, 6765, 6770, 6771, [MUSM]; 1 male: same data, except 12.vi.2000, R.K. Robbins, MUSM-ENT-006769, [MUSM]; 1 female: Llacanora, [7°11′S,78°25′W], 2720 m, 19.xii.2006, [R. Vila], [MUSM]; 1 male: Limón-Santa Rosa, 1800–3000 m, i-ii.1998, R. Marx, FLMNH-MGCL-147145, [FLMNH]; 1 male: same data, FLMNH-MGCL-147146, [FLMNH]; 1 male: same data, FLMNH-MGCL-147147, [FLMNH]; 1 male: same data, FLMNH-MGCL-147148; [FLMNH]; 1 male: same data, FLMNH-MGCL-147149; [FLMNH];1 male: same data, FLMNH-MGCL-147150, [FLMNH]; 1 male: vía Celendín-Balsas, El Choloque, [6°51′57″S,78°4′35″W], 1800–2000 m a.s.l., local collectors, vi-vii.2006, [MABO]; *Amazonas*: 1 male: Chachapoyas, [06°10′S,77°38′W], [2343 m], 1889, M. de Mathan, Ex Oberthür Coll. Brit. Mus. 1927–3., MUSM-ENT-006766, [MUSM]; 1 male: Molinopampa—Granada, [06°23′S,77°26′W, 2800–3000 m a.s.l.], B. Calderón leg., [CEP-UJ]; ECUADOR: *Azuay*: 1 male: Oña, [03°28′27″S,79°9′32″W], 2200 m a.s.l., 22.iii.[19]65, L.E. Peña, [MUSM].

*Diagnosis: Phulia phantasma* n. sp. can be recognized from species of similar size previously placed in *Hypsochila*, *Tatochila*, or *Theochila* by its more compact appearance resulting from its less elongate wings, being similar in this respect to some species of *Hesperocharis* C. Felder, 1862, such as *H. marchalii* (Guérin-Méneville, [1844]), which is actually syntopic with *P. phantasma* n. sp. These two species of similar size and flight pattern can be easily confused in the field. Particularly pale individuals of *P. phantasma* with reduced black in the DFW apex and reduced (or almost absent) dark VHW markings are very similar to some individuals of *P. maenacte* from southeastern Brazil, but can be readily distinguished (apart from distribution) by having the base of vein *R*_4+5_ on the FW much closer to the distal margin than it is to the base of vein M_1_, whereas in *P. maenacte* it is approximately equidistant. Otherwise, *P. phantasma* n. sp. can be distinguished from *P. maenacte* by the dark scaling that lines the VHW veins broadening and fusing towards the distal margin, rather than tapering or remaining similar in width, and by the presence of a line of dark postdiscal spots in cells M_1_-Rs to CuA_2_-2A on the HWV, which may be shaped like distally pointing arrows, or just present as diffuse dark scaling, always absent in *P. maenacte* but similar to species previously placed in *Hypsochila* or several species of *Tatochila*. From the majority of other species of *Phulia*, *P. phantasma* n. sp. may be distinguished by the lack of any dark scaling at the end of the FW discal cell, with the dark markings in the distal half of the FW present as a simple black triangular patch which variably fills the apex and may be almost absent. Other superficially similar species (such as those formerly placed in *Hypsochila*, e.g., *P. microdice* or *P. huemul* (Peña, 1964)), have at least the FW discocellular veins lined with black, and a variable line of black FW postdiscal spots.

**Fig. 6:**
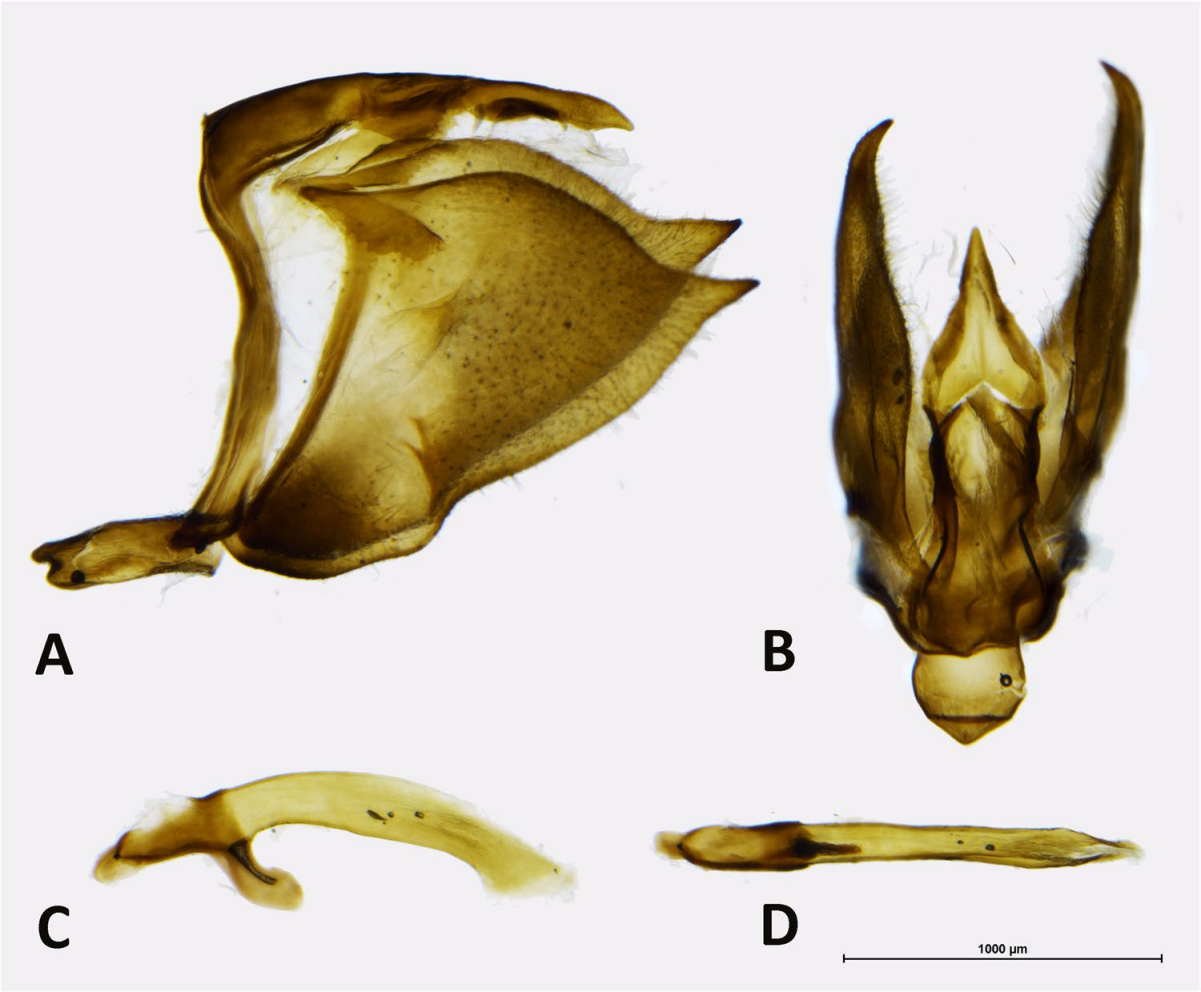
Male genitalia of *Phulia phantasma* n. sp.: **A** lateral view; **B** vertical view; **C** aedeagus lateral view; **D** aedeagus vertical view

*Description*: MALE (Fig. 3A–B): *Head*: eyes brown, bare, with narrow fringe of white scales at base and a yellow dorso-lateral spot; antennal shaft mixed black and white dorsally (24 antennomeres) with conspicuous rounded club (8 antennomeres) which is pale yellow except for proximal dorsal half which is black; labial palpi white with sparse long, black hair-like scales; top of head and frons white. *Thorax*: dorsal surface with black scales and long white hair-like scales from sides and near wing bases, ventral surface white, legs with sparse white scaling except for femur with denser white scaling and long white hair-like scales, mid- and hindlegs lacking tibial spurs (present in *P. maenacte*; Field 1958). *Wings*: Forewing (length 27–28 mm, *n* = 5) triangular, hindwing an elongate oval with slightly straighter margin in middle of wing and angled tornus; FW with four radial veins, *R*_4_ and *R*_5_ fused, with veins *R*_4+5_ and *R*_3_ originating relatively close to the apex (*R*_4+5_ approximately half length of *R*_3+4+5_), *M*_2_ originating independently of base of *M*_1_ + *R*_3+4+5_. FWD ground color white, variably present scattered blackish scaling filling apex with uneven basal edge indented in middle of each cell, scattered blackish scaling at very base of wing. HWD similar to forewing except lacking black apical scaling. FWV similar to dorsal surface except lacking dark scaling at wing base and dark apical scaling slightly paler, tinged yellowish, with very indistinct paler yellowish scaling forming intervenal stripes within darker apical area, costa with scattered pale grayish scales. HWV ground color pale yellow, slightly darker orange-yellow anterior of discal cell and more prominently along edge of costa, dark orange-yellow spot at base cell 2A-Cu_2_; scattered dark grayish scaling lining edges of veins and a forming a “Y”-shaped marking in middle of discal cell, line of indistinct dark gray postdiscal markings in cells 2A-Cu_2_ to Rs varying from distally pointing arrow shapes to indistinct spots, grayish scaling lining veins broadening and fusing towards margin. *Abdomen*: dorsal surface black, ventral surface white. *Genitalia* (Fig. 6): similar in overall form to other members of the genus, for example *Phulia wagenknechti* (type species of *Hypsochila*) as figured by Field (1958), notable features include long uncus (similar in length to tegumen), valvae with a blunt distal point, aedeagus of even width, and opening ventrally. In comparison with *P. maenacte*, there are several differences in the male genitalia, including in *P. phantasma* n. sp. a much longer uncus, greatly reduced in *P. maenacte*, and a tapering, downward-pointing aedeagus (flaring and upward-pointing in *P. maenacte*). FEMALE (Fig. 3C–D): similar to male, except as follows: forewing (length 25 mm, *n* = 1) and hindwing more rounded, ground color slightly yellowish, dark DFW apical marking more extensive and with intruding pale scaling middle of each cell; ventrally similar to heavily patterned males, FW with trace of indistinct yellowish lines in middle of each cell in apical half, HW with stronger yellowish tinge to pale areas. Genitalia not examined.

**Fig. 7:**
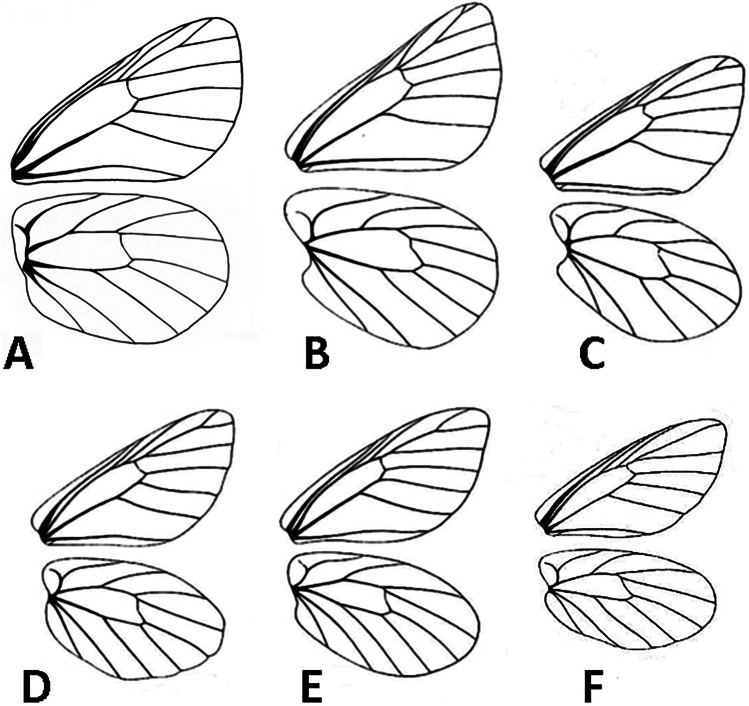
Venation pattern of *Phulia*. **A** *Phulia stoddardi* n. sp.: **B** *Phulia* (*Piercolias*) *huanaco*; C. *Phulia* (*Pierphulia*) *nysias*; **D** *Phulia nymphula*; **E** *Phulia garleppi*; **F** *Phulia* (*Infraphulia*) *madeleinea* (B-F redrawn from Field, 1958)

*Etymology*: The name is a neuter Latin noun in the nominative singular meaning a ghost or phantom, in reference to the pale markings of this species and its mysterious rarity in collections.

*Bionomics and distribution*: This species is known from a large area of the central tropical Andes, extending from southern Ecuador to central Peru (Fig. 13). It is rare in collections which does not reflect its status in the field. The perceived rarity results from this species occurring in the areas which are seldom visited by lepidopterists, dry Andean valleys, and especially being confused with common white pierids, especially with *Hesperocharis marchalii* and *Leptophobia aripa* (Boisduval, 1836). Additionally, *P. phantasma* n. sp. is a very active, patrolling butterfly, only sporadically seen visiting flowers, such as of yellow and purple *Onoseris albicans* (Asteraceae) flowers (Fig. 12E), and not seeking humid areas where mud-puddling other pierids are frequently found. The flight is fast and swift. Although it has been recorded from a wide altitudinal range from 1200 to 4131 m, it seems that its optimal elevational range is between 2000 and 2800 m a.s.l. All individuals were collected between December and July, representing the wet season and first half of the dry season.

### Molecular phylogeny

**Fig. 8:**
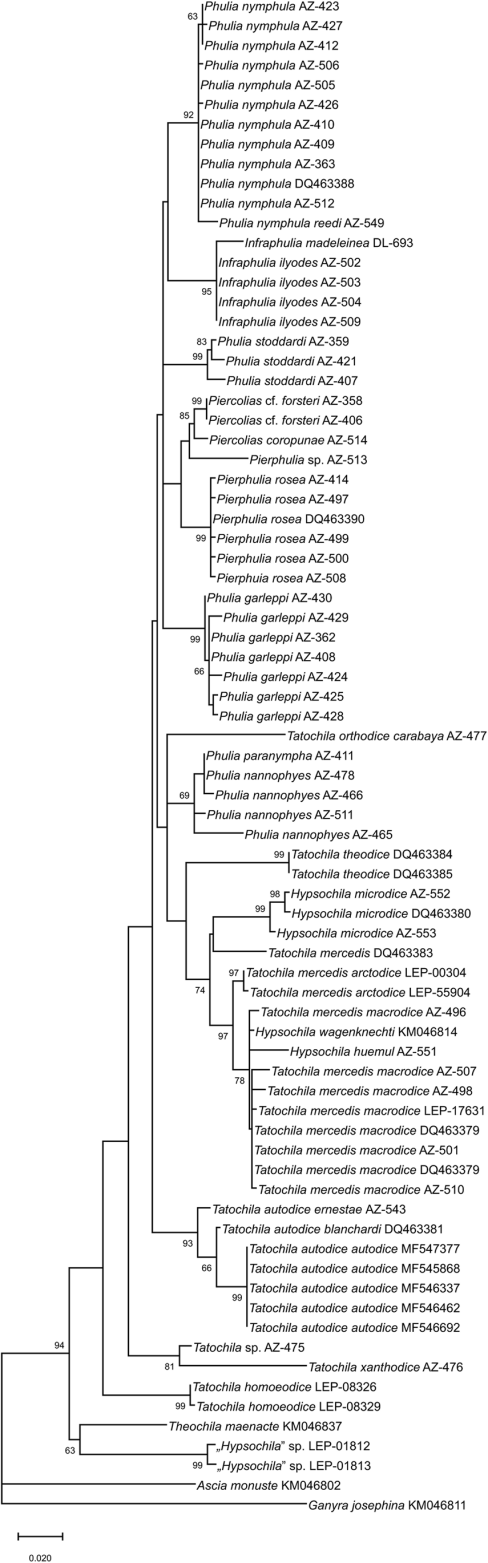
COI ML tree of *Phulia*, bootstrap values above 60 indicated at nodes. All the generic names used previously are shown in order to underline their relative position in the phylogeny. The genera *Ascia* and *Ganyra* are used as outgroups

**Fig. 9:**
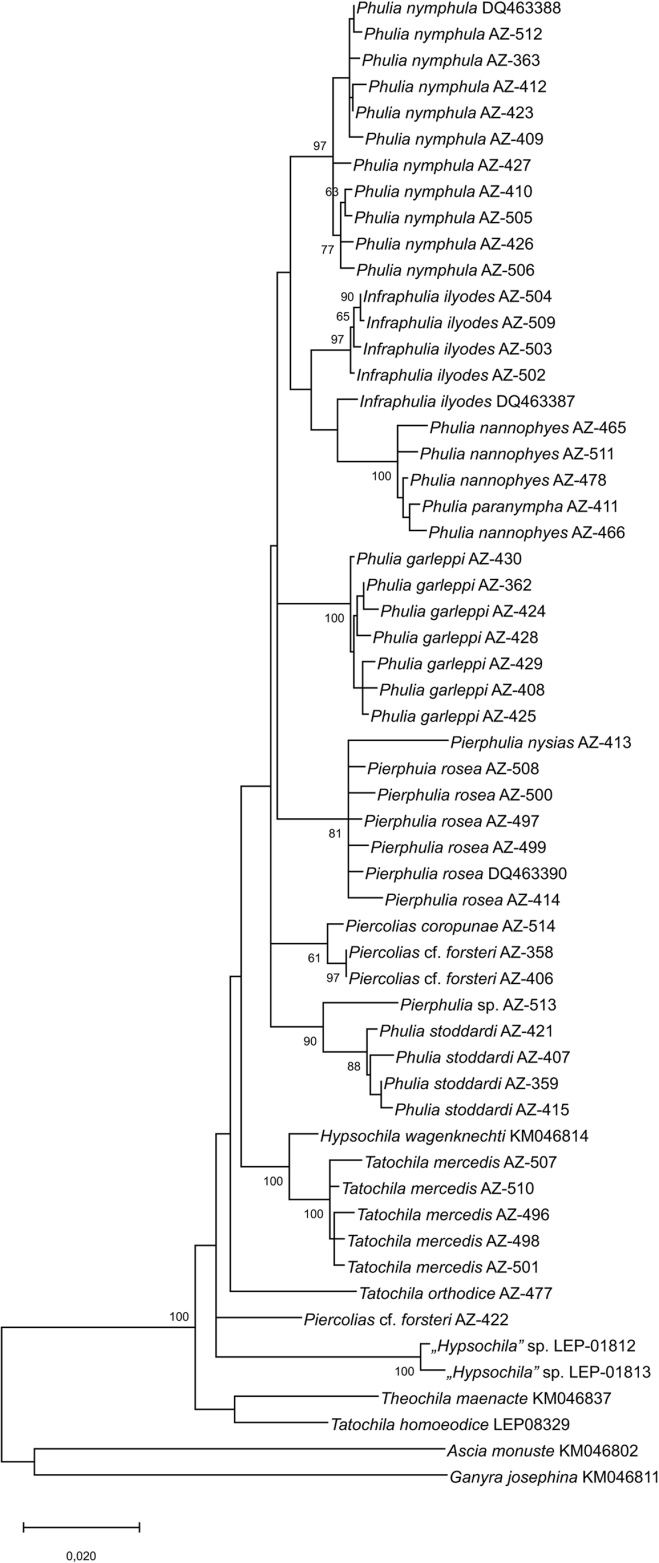
Concatenated (COI, GAPDH, RpS-5) ML tree of *Phulia*, bootstrap values above 60 on the nodes. All the generic names used before are shown in order to underline their relative position in the phylogeny. The genera *Ascia* and *Ganyra* are used as outgroups

Our study provides strong support for the monophyly of the expanded concept (Zhang et al. 2021) of *Phulia* in both the COI (bs: 94) (Fig. 8) and the concatenated 3-genes tree (bs: 100) (Fig. 9). The internal topology of this large clade differs, however, between analyses based on only COI or on all three genes.

In the COI tree, former *Phulia* cluster with former *Piercolias*, *Pierphulia*, and *Infraphulia*, as well as with former *Hypsochila* and three species formerly placed in *Tatochila*, *P. orthodice* (Weymer & Maassen 1890)*, P. mercedis*, and *P. theodice*, and forms a sister clade to *P. autodice*. *Phulia xanthodice* (Lucas 1852) and *Phulia* sp., in turn, form a sister clade to former *Phulia* and other species plus *P. autodice*, whereas *P. homoeodice* is situated on a long external branch relative to all previously mentioned species. Finally, *Phulia maenacte* and *Phulia phantasma* cluster together and are sister to all the other taxa. However, the COI tree is not fully resolved because the support values on deeper nodes are low. On the other hand, the support of terminal branches is high but they refer only to single, or exceptionally two species, simply meaning that the species identification based on external morphology fully agrees with barcode data.

In the concatenated tree, the former genera *Phulia*, *Pierphulia*, *Piercolias*, and *Infraphulia* cluster together in a weakly supported clade (bs: 41), sister to *Phulia mercedis* + *Phulia wagenknechti*, but branch support is low (bs: 48). *Phulia orthodice* is situated in an external position relative to all the abovementioned taxa, on a long branch, but again the common node support is low (bs: 36). Finally, *Phulia maenacte* and *Phulia phantasma* n. sp. form a weakly supported clade sister to all the other taxa. As in the COI tree, all the small internal clades composed of one or two species are strongly supported. In the concatenated tree, only a few species of former *Tatochila* + *Hypsochila* were included, which hampers understanding of the relationships of species formerly included in these genera in relation to other Pierina, and in particular the species of the *Phulia* sensu stricto clade. The only interesting noticeable difference is the position of *Phulia orthodice* as sister to the *Phulia s. s.* clade on the concatenated tree, and well-rooted in the *Phulia* sensu stricto clade in the COI tree. Otherwise, the topology of both trees is roughly similar, in particular the sister status of the *Phulia phantasma* n. sp. + *Phulia maenacte* clade in relation to the remaining genera.

**Fig. 10:**
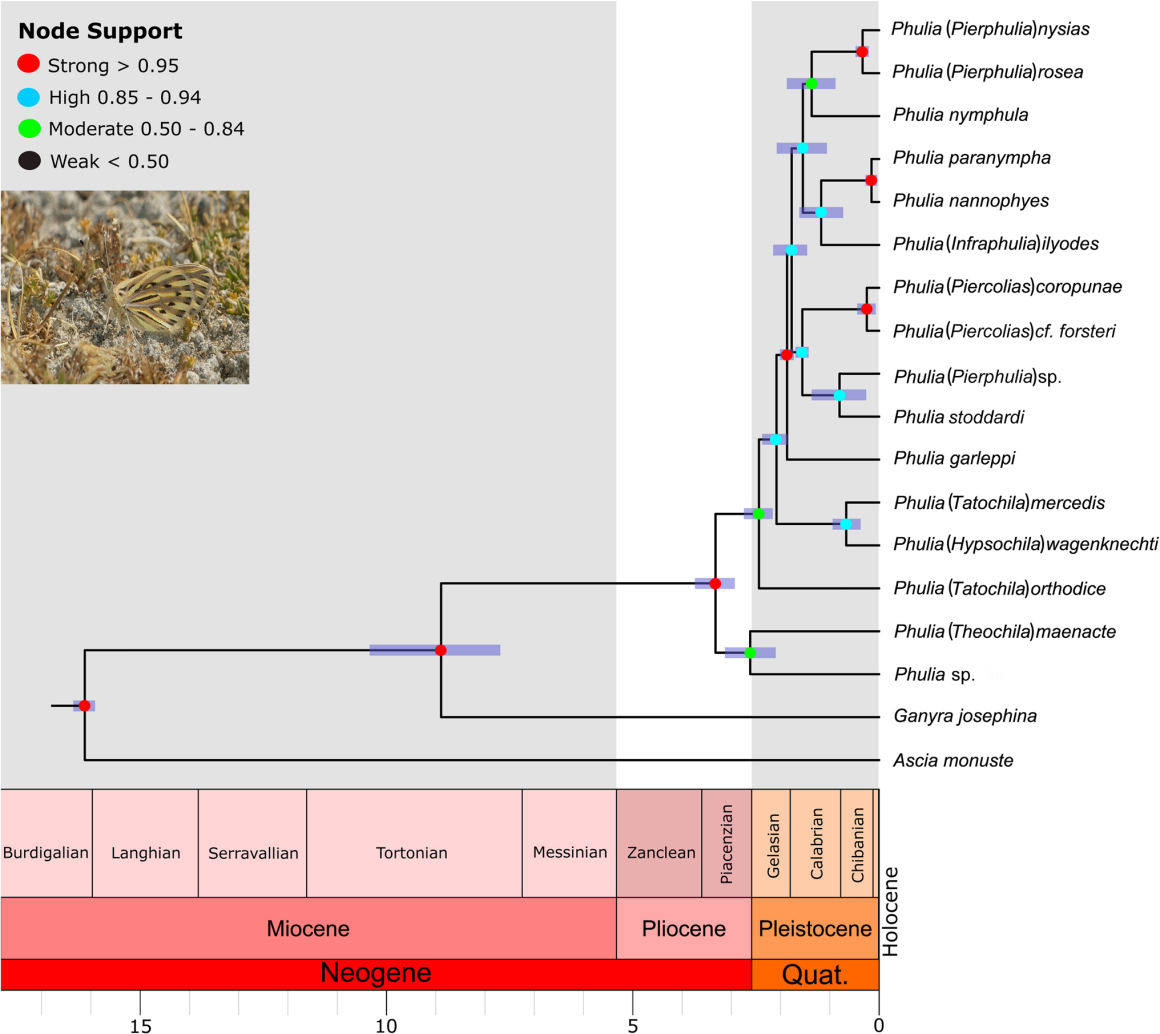
A hypothesis of divergence timing of the genus *Phulia* (generic names used previously in parenthesis)

Our results infer a divergence of the *Phulia* sensu lato clade from *Ganyra josephina* in the middle Miocene (Tortonian), ~ 8.8 Mya (4.97–11.8 HPD 95%) (Fig. 10), followed by the subsequent divergence of the two main clades of the *Phulia* group in the late Pliocene (Piacenzian), ~ 3.3 Mya (2.4–4.2 HPD 95%). The radiation of *Phulia* sensu lato occurred throughout the Pleistocene, with the divergence of *P. maenacte* and *P. phantasma* n. sp. at ~ 2.5 Mya (1.72–3.03 HPD 95%), and that of other clades of *Phulia* sensu lato slightly later, at ~ 2.1 Mya (1.55–2.51 HPD 95%). The two clades which form the *Phulia* sensu stricto group originated at ~ 1.8 Mya (1.34–2.07 HPD 95%). In addition, *Phulia garleppi* diverged from the rest of *Phulia* sensu lato at ~ 1.9 Mya (1.46–2.18 HPD 95%). The clades (*Phulia stoddardi* n. sp. + *Phulia* (“*Pierphulia*”) sp.) + two species previously associated with *Piercolias* (*P. cf. forsteri* + *P. coropunae*)) diverged at ~ 1.6 Mya (0.52–1.88 HPD 95%). The group comprising two species previously associated with *Pierphulia* (*P. rosea* + *P. nysias* (Weymer & Maassen, 1890)) + *Phulia nymphula*) separated at ~ 1.4 Mya (0.39–1.62 HPD 95%). The group comprising the species previously placed in *Infraphulia* (*P. ilyodes*) + *Phulia* sensu stricto (*P. nannophyes* Dyar, 1913 + *P. paranympha* Staudinger, 1894)) at ~ 1.2 Mya (0.36–1.51 HPD 95%), and the clades ((*P. rosea* + *P. nysias*) + *P. nymphula*) + (*P. ilyodes* + (*P. nannophyes* + *P. paranympha*)) diverged at ~ 1.5 Mya (1.1–1.83 HPD 95%) (Fig. 10).

**Fig. 11:**
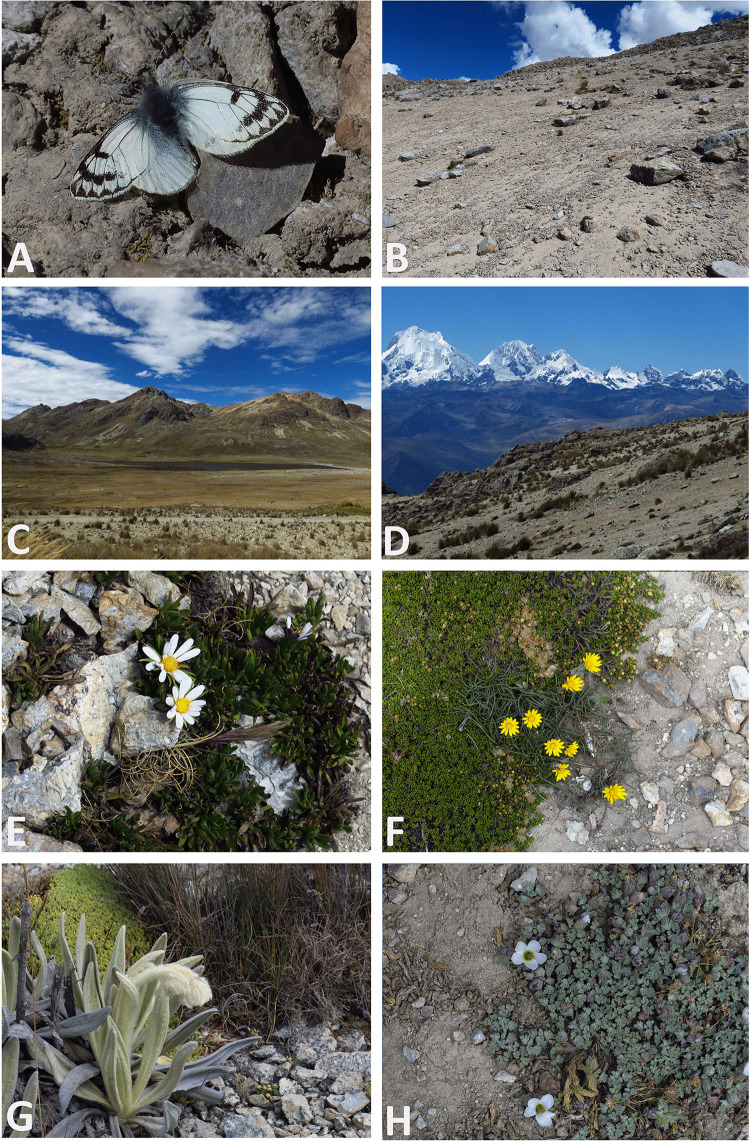
Habitat of *Phulia stoddardi* n. sp. in type locality: **A** *Phulia stoddardi* n. sp. male in the field, thermoregulating on rocky substrate; **B** sandy slope with lose boulders where most observations of adults took place, 4850–4900 m; **C** view on the lake and boggy area at 4700 m, biotope of *Phulia garleppi* were abundant; **D** view of the Cordillera Huaywash; **E** *Werneria* sp., and Asteraceae sp.; **F** Asteraceae sp.; **G** *Senecio* sp.; **H** *Gentiana sedifolia* (photos: P. Boyer)

**Fig. 12:**
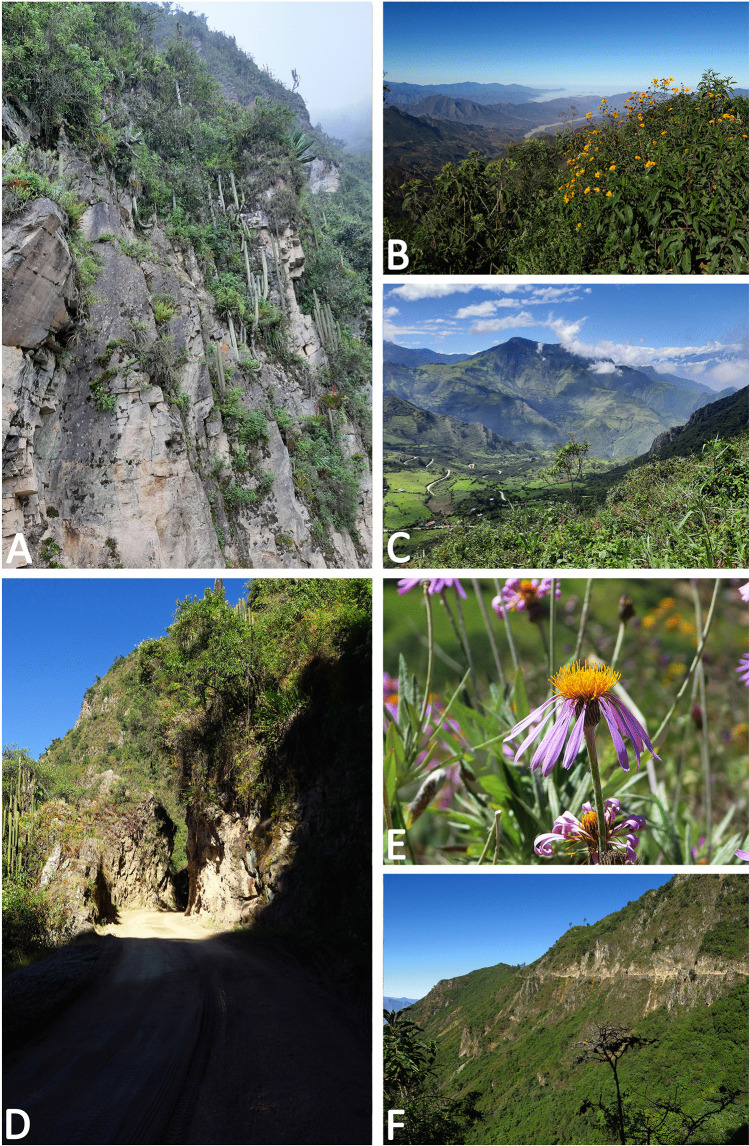
Habitat of *Phulia phantasma* n. sp.: A. A cliff above the Contumazá – Cascas road B. Asteraceae plants along the road; **C** view on the valley of Cascas; **D** xerophytic vegetation along the Contumazá–Cascas road, 2700 m; **E** flowers of *Onoseris albicans* (Asteraceae) frequently visited by numerous species of pierids, including *P. phantasma* n. sp.; **F** patches of cloud forest below the Contumazá–Cascas road, 2700 m (photos: P. Boyer)

**Fig. 13:**
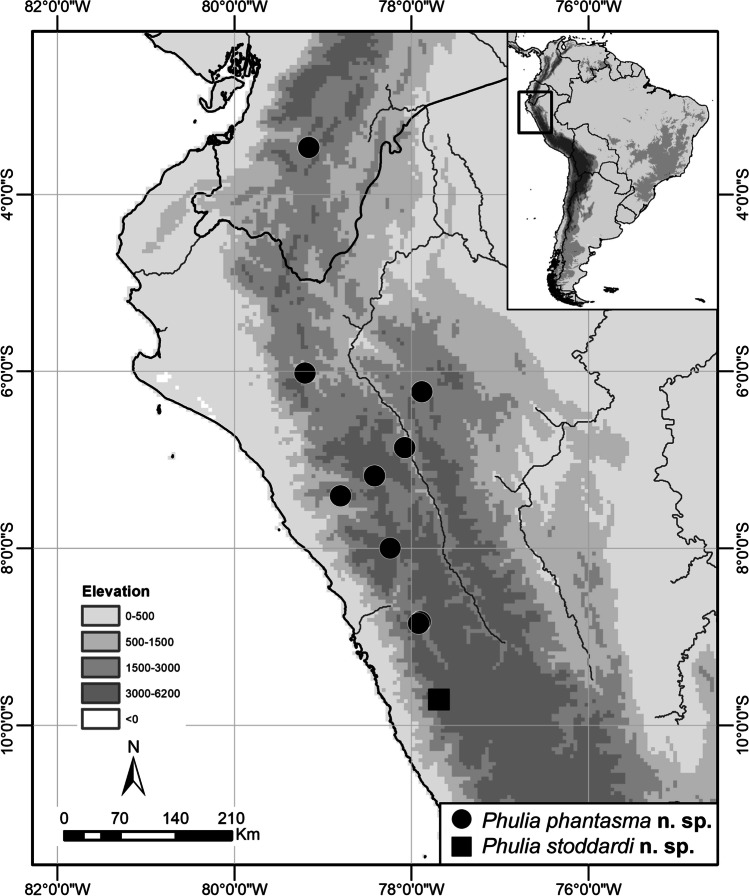
Distribution of *Phulia phantasma* n. sp. and *Phulia stoddardi* n. sp

## Discussion

Our results broadly corroborate those of the global genomic study of butterflies of Zhang et al. (2021) and specifically the proposed synonymy of most of the genera (except the loosely related *Leptohobia*) of montane/temperate Neotropical Pierina with the genus *Phulia*. The results of that study, however, must be considered preliminary given that although most extant genera were examined, only 11 species were included, and just one or exceptionally two, species per genus. This sample size is potentially too small to reach definitive conclusions on the internal topology of the Neotropical Pierina clade and hence to develop the most appropriate classification. Our study aimed to broaden the taxon sampling, and although it was based on only three genetic markers, it included a much more comprehensive set of taxa, involving 73 samples of almost all known species within this clade (except for two assigned previously to *Tatochila* and of doubtful specific status (*T. inversa* Hayward, 1949, and *T. mariae* Herrera, 1970), and two formerly placed in *Hypsochila*).

What is clear from both our sets of data is that retaining the systematic status quo is not tenable. This conclusion applies in particular to the retention of *Infraphulia*, *Pierphulia*, and *Piercolias* as separate from *Phulia*. Such a decision would require the description of more new, species-poor or monobasic genera, for *Phulia garleppi* and *Phulia stoddardi* n. sp., without resulting in genera that are morphologically or ecologically reconizable.

The expansion of the former genus *Phulia* by adding the above three former genera seems appropriate. On the concatenated tree, the clade *Phulia* sensu stricto plus those three former genera was monophyletic and sister to *P. mercedis* + *P. wagenknechti*. However, the clade has a weak node support, and several species assigned before to *Tatochila* were not included. On the COI tree, on the other hand, the *Phulia* sensu stricto plus those three former genera was evidently paraphyletic and was recovered as monophyletic only through the addition of several species previously assigned to *Tatochila*, in particular *P. orthodice*, *P. theodice*, *P. mercedis*, and also both species formerly placed in *Hypsochila*. Further expanding the genus *Phulia* to include several considerably larger species included before under *Tatochila* would require a totally new generic diagnosis. In fact, the genus *Phulia*, apart from some minor features of venation pattern, was previously differentiated from *Tatochila* mostly, if not only by, its considerably smaller size (Field 1958).

For similar reasons, maintaining the separate generic status of *Tatochila* is not defensible. This taxon as formerly conceived is clearly not monophyletic, with some species clustering either with *Phulia* sensu stricto and relatives, or situated in an external position in relation to *Phulia* along with some species of former *Tatochila*, in particular *P. orthodice* on the concatenated tree, and *P. xanthodice*, *P.* sp., and *P. homoeodice* on the COI tree. In fact, in the light of our molecular study, the retention of *Tatochila* would require restricting it to the nominate species *Synchloë autodice*, while removing *P. mercedis* + former *Hypsochila* to the expanded *Phulia*, and describing new genera for *P. xanthodice* and *P. homoeodice*. Even acknowledging that *T. homoeodice*, in particular, is morphologically quite different from other species of former *Tatochila*, the redefining and retaining of *Tatochila* would cause significant systematic chaos without resulting in readily recognizable or ecologically compact genera.

The synonymy of all the above genera is further supported by our analysis which indicates a very recent divergence time for the entire group, which would have occurred throughout the Pleistocene, and is correlated with the timing of the Andean orogeny and the availability of high-elevation biomes which developed in the Pliocene some 3–5 Mya (Gregory-Wodzicki 2000). Major climate oscillations in the Pleistocene resulting in subsequent ice ages and warmer interglacials are thought to have promoted habitat turnover and heterogeneity, contributing to faster rates of speciation (Nevado et al. 2018); the timing of the radiation of *Phulia* is congruent with the massive radiation of páramo plants, such as the megadiverse genus *Hypericum*, the overwhelming majority of which occurred in the Pleistocene (Nürk et al. 2013).

Finally, the generic classification of *Phulia maenacte* (Fig. 3E–H) and *Phulia phantasma* n. sp. would be problematic if attempts were made to retain some of the genera discussed above. These two species diverged from the remaining taxa of the *Phulia* clade at a somewhat earlier stage, in the Pliocene. The node of the two taxa is weakly supported and their relative branches are long. *Theochila* is endemic to southeastern Brazil, marginally penetrating into northern Paraguay and Argentina, where it occurs mostly in pampa grasslands from low elevations above the sea level up to 2200 m a.s.l., whereas *Phulia phantasma* n. sp. is a mid- to high-elevation xeric shrub specialist with adults markedly different from other Andean Pierina. Merging the two species within *Theochila* would not be justified from any point of view, molecular, biogeographic, or morphological. Their retention as separate entities, on the other hand, would require the description of a second, monobasic genus for *Phulia phantasma* n. sp., also an unhelpful decision for constructing a useful classification. The external position of *Phulia homoeodice* in relation to all other taxa of the *Phulia* complex, including the above two on the COI ML tree, is unexpected. We advocate, at this stage, to maintain Zhang et al.’s (2021) synonymy of all these generic names with *Phulia*, pending a more comprehensive molecular study of the entire Pierina using substantial DNA sequence data.

The discovery of a new extreme high-elevation species of *Phulia* in central Peru is a timely reminder that our knowledge of the butterfly fauna of the highest elevations of the Andes remains superficial, and that we still have much to learn about the evolution and biogeography of Andean butterflies. First of all, the elevations at which the species associated previously with *Piercolias* and the new *P. stoddardi* n. sp. occur are seldom visited by entomologists, and even when they are, the probabilities of discovering a population of these species remain very low. Their flight period is, as far as is known, confined to the hottest and driest seasons, as for most of the year at 5000 m a.s.l. temperatures remain very low even during the day, with snowfall and constant cloud cover. The difficulty of finding these butterflies on the wing is emphasized by the recent discovery of *Phulia* (*Piercolias*) cf. *forsteri* in Ticlio (Paso Anticona), one of the most frequently visited but seldom collected places in Peru, situated on the main road from Lima to the Amazon basin, and whose Pierinae were briefly studied by Shapiro (1985).

The physiological challenges that this new species needs to overcome, to be able to survive in the climatic conditions at 5000 m a.s.l. in the Andes, are quite unparalleled. Although many Arctic butterflies face very low temperatures, these typically happen during their early stages, in most cases as larvae or pupae, which can find shelter in the soil, leaf litter or under stones, and are therefore much more resistant to extremely low temperatures. In this case, however, the adults also have to survive extreme daily temperature changes. During the hottest days, for example, in Ticlio at 4800 m a.s.l., the temperature can rise to 16–17 °C, and the surface of objects, where the butterflies like to sit, such as rocks, sand, and organic matter, can reach substantially higher temperatures in the strong sun, whereas minimum night temperatures drop to − 20 °C, implying a range of temperature within a 24-h period of around 40 °C. This diurnal range is matched nowhere else, even in Arctic areas or in the mountains of Central Asia. At 5000 m a.s.l. in the Himalayas, maximum day temperatures in July–August are around 12 °C, and night just below 0 °C (www.brittanica.com). Adults of *P. stoddardi* n. sp. face not only extremely low temperatures but also desiccation since their habitat is very dry and low humidity is reinforced by extreme winds. Gusty winds, which blow for most of the day, can potentially make any insect, and butterfly in particular, flight activity energetically costly or impossible (Mani 1968).

It is also worth noting that most other species of this group, previously assigned to *Phulia*, *Infraphulia*, and *Pierphulia*, are found associated with high-altitude bogs and remain close to humid areas with a predilection for resting on mossy, wet surfaces, or among low grasses, even if they are seen occasionally in drier areas (Shapiro 1985). They can occasionally be observed overflying screes and dry gullies away from marshes, which may be a result of high population densities with some individuals tending to disperse over long distances. However, *P. stoddardi* n. sp., as well as those species associated before with the genus *Piercolias*, are highly specialized to very dry, well-exposed sandy slopes with sparse stones and loose cushion plants. This new species also appears to be restricted to a narrow altitude band since all the individuals were observed between 4750 and 5050 m a.s.l., whereas other local species of *Phulia* or *Colias*, while occasionally observed near 5000 m a.s.l., have preferred habitats at lower elevations—a phenomenon also found in Central Asia, where some species of *Baltia* or *Pontia* may occur locally up to 5400 m a.s.l., but can equally be found down to 3500 m a.s.l.

A detailed study of the ecology and biology of *P. stoddardi* n. sp. is therefore highly desirable from the perspective of its presumably unique adaptations to its unusual environment. Interestingly, there is apparently no wing size reduction in *P. stoddardi* n. sp., nor those species associated previously with *Piercolias*, as a presumed adaptation to extreme altitude environments. On the contrary, these species are of considerably larger size than their close relatives placed previously in *Phulia*, *Infraphulia*, and *Pierphulia*, which also generally occur at lower elevations.

Larval host plants and early stages are known for several species assigned previously to *Tatochila* and *Hypsochila*, which feed on Fabaceae (Shapiro 1990, 1991) and Brassicaceae (Shapiro 1985, 1990; Olivares and Hormazábal 1998) but also on Tropaeolaceae (Shapiro 1990). Reports of *Tatochila* feeding on secondary *Cestrum* Solanaceae were questioned (Shapiro 1979). *Phulia xanthodice* is observed frequently in Venezuela and Colombia flying in cabbage fields, which is most probably its substitute host plant. On the other hand, natural hostplants of extreme high-altitude Pierina are unknown. In laboratory conditions *Phulia* (*Pierphulia*) *rosea* was found to lay eggs and feed on the small *Lepidium virginicum* (L.) Brassicaceae (Shapiro 1986), and the first-instar larvae of *Phulia* (*Infraphulia*) *ilyodes* was reported to accept *Brassica nigra* (L.) (Shapiro 1985). There is information that, at least in Argentina, *Phulia nymphula* feeds on *Tropaeolum polyphyllum* Cav. (Reed 1918), but this is very unlikely in the areas where *Phulia stoddardi* n. sp. occurs in Peru since no Tropaeolaceae were seen, and these plants usually occur at rather lower elevations. There are very few candidate plants as hosts for *Phulia stoddardi* n. sp., for example, *Draba* spp. which are one of the few Brassicaceae known to occur at near 5000 m a.s.l. However, no species of *Draba* were spotted in the area where adults of *Phulia* were flying.

An underlying question is: are there more extreme high-altitude butterflies to be discovered in the Andes? Very likely so. The discovery of this new species in the department of Ancash leads to the conclusion that the fauna of extreme altitude pierids is far from being well known and that the exploration of other areas with similar ecological conditions in Peru, Bolivia, or even northern Chile or Argentina could lead to the discovery of yet other fascinating species, or even genera. How high do these species occur? We have found them around 5000 m a.s.l., but, admittedly, higher elevations have not been explored, and there are apparently appropriate habitats at 5300–5500 m a.s.l. in the nearby Cordillera de Huayhuash.
