# Correction: Considerations on the Systematics of Neotropical Pierina, with the Description of Two New Species of *Phulia* Herrich-Schäffer from the Peruvian Andes (Lepidoptera: Pieridae, Pierinae, Pierini)

**DOI:** 10.1007/s13744-023-01045-1

**Published:** 2023-04-26

**Authors:** Tomasz W. Pyrcz, Keith R. Willmott, Gerardo Lamas, Pierre Boyer, Klaudia Florczyk, Christer Fåhraeus, Oscar Mahecha, José Cerdeña, Artur Mrozek, Jackie Farfán, Anna Zubek

**Affiliations:** 1 Dept of Invertebrate Evolution, Institute of Zoology and Biomedical Research, Jagiellonian Univ, Kraków, Poland; 2 McGuire Center for Lepidoptera and Biodiversity, Florida Museum of Natural History, Univ of Florida, Gainesville, FL USA; 3 Museo de Historia Natural, Univ Nacional Mayor de San Marcos, Lima, Perú; 4 Le Puy Sainte Réparade, France; 5 Nature Education Centre of the Jagiellonian Univ, Kraków, Poland; 6 Dept of Biology, Lund Univ, Lund, Sweden; 7 Grupo en Biogeografía y Ecología Evolutiva Neotropical BEEN, Univ Distrital F.J.C./Univ Incca de Colombia, Bogotá, Colombia; 8 Museo de Historia Natural, Escuela de Biología UNSA, Univ Nacional de San Agustín de Arequipa, Arequipa, Perú

**Correction: Neotropical Entomology (2022) 51:840–859**

**https://doi.org/10.1007/s13744-022-00999-y**

The ZooBank registration was missing in the original article and is published here. The original article has been updated. New taxa published in this article will have the date of this correction as their date of publication.

ZooBank Registration: urn:lsid:zoobank.org:pub:B5FC1030-BEFF-4F68-B6C5-6B47495BFC2F.

